# Gambling Before and During the COVID-19 Pandemic Among Online Casino Gamblers: An Empirical Study Using Behavioral Tracking Data

**DOI:** 10.1007/s11469-020-00462-2

**Published:** 2021-02-02

**Authors:** Michael Auer, Mark D. Griffiths

**Affiliations:** 1 neccton Ltd., Muhlgasse 23, 9900 Lienz, Austria; 2 International Gaming Research Unit, Psychology Department, Nottingham Trent University, 50 Shakespeare Street, Nottingham, NG1 4FQ UK

**Keywords:** COVID-19, Gambling, Online gambling, Casino gambling, Behavioral tracking

## Abstract

Gambling, like many other leisure activities, has been greatly affected by the novel coronavirus disease 2019 (COVID-19) pandemic. The present study investigated the behavior of a sample of online casino gamblers before and after the COVID-19 pandemic was announced in March 2020. The authors were given access to behavioral tracking data of a representative sample of 133,286 online casino gamblers by a large European online gambling operator with several online casino Swedish licenses. Online casino gambling activity utilizing daily cross-sectional data was examined over a 5-month period from January 1 to May 31 (2020). Results indicated that the (i) number of active online casino gamblers significantly increased over time, (ii) mean average amount of money bet by online casino gamblers daily significantly decreased over time, (iii) mean average daily bet by online casino gamblers at both the 90th and 99th percentiles significantly decreased over time, and (iv) mean average daily bet by online casino gamblers at the 10th and 25th percentiles significantly increased over time. The analysis also indicated that the number of high-risk players significantly decreased during the 5-month study period. While many different groups have claimed that gambling and problem gambling would increase during the pandemic due to more time being spent at home, evidence from the present study suggests that this is not the case because gambling intensity decreased, at least among Swedish gamblers.

The novel coronavirus disease 2019 (COVID-19) pandemic has resulted in many changes to individual’s daily lives (e.g., national lockdowns, spatial distancing, home quarantine, and no large gatherings of individuals) (Pakpour and Griffiths 2020). For gamblers, one of the most salient consequences was that all major sporting events were postponed or canceled for a number of months meaning that such events could not be bet upon (Auer, Malischnig and Griffiths 2020). This led to speculation by various stakeholders (e.g., treatment service providers, policymakers, gambling regulators, and scholars) whether sports bettors would substitute their betting with other forms of gambling and whether there would be an increase in online gambling more generally because individuals were spending long periods at home with lots of spare time to fill. Given the fact that COVID-19 was only declared as a world pandemic in March 2020, there has been relatively little research examining its effects on gambling behavior.

Price (2020) surveyed 2005 Canadian gamblers during the first 6 weeks of lockdown measures and assessed problem gambling using the Problem Gambling Severity Index as well as other factors including mental health concerns, financial impacts due to the pandemic, substance use, and gambling motivations. Results on a subsample of 1081 online gamblers indicated that high-risk gamblers among this group had an increased likelihood of gambling during the Canadian lockdown and that one of the predictive risk factors for high-risk online gamblers included the influence of COVID-19 (in response to the statement “Emergency measures implemented due to COVID-19 influenced my decision to gamble online over the past 6 weeks”). Other risk factors included severe depression and anxiety and gambling while drinking alcohol or smoking cannabis. Price concluded that the study “confirmed many of the risk associations presented in emerging COVID-19-related studies and past research on global economic crisis relating to gambling risk, mental health concerns and substance use” (p. 1).

Håkansson (2020) collected self-reported information from Swedish participants (*n* = 2016) about their online gambling involvement during the pandemic. The study concluded that only a minority of participants reported increased gambling in response to the pandemic, but this group had markedly higher gambling problems and increased alcohol consumption and may represent a subgroup with a particularly high vulnerability. Additionally, Håkansson did not find increased gambling among the high-risk and most intense online casino players. However, self-reported spend on gambling does not necessarily reflect actual gambling. Auer and Griffiths (2017) compared actual gambling spend (using behavioral tracking data) with self-reported data on gambling spend from the same players and found that there were significant differences between self-reported gambling intensity and actual gambling intensity. More specifically, regular gamblers underestimated their losses and overestimated their wins.

In the gambling studies field, there has been an increasing use of behavioral tracking data over the past 15 years (Griffiths 2020). While the aforementioned survey provides potentially useful data, self-reported surveys and other self-reported methodologies are subject to common method biases (most notably poor memory recall and social desirability). However, account-based tracking data provides objective and accurate data about gambling behavior (e.g., Auer and Griffiths, 2014, 2015, 2016, 2017, 2020; Braverman et al. 2013; Braverman and Shaffer 2012; Broda et al. 2008; Dragicevic et al. 2015; LaBrie et al. 2008, LaPlante et al. 2008, 2009; Leino et al. 2015, 2017; Nelson et al. 2008; Sagoe et al. 2018; Xuan and Shaffer 2009). Additionally, the sample sizes tend to be much larger than other methodologies such as surveys, experiments, and qualitative studies (e.g., focus groups and interviews).

A recent study by Auer, Malischnig, and Griffiths (2020) using tracking data supplied by a European gaming operator found that the amount of money bet by online sports bettors significantly decreased during the COVID-19 pandemic (although this was expected given that there was little sport to bet on during the early stages of the pandemic). The study also found that online sports bettors did not substitute their sports betting with online casino playing. The authors first classified sports bettors into ten different groups according to the number of distinct weeks with at least one wager. In all of the groups, the percentage of sports bettors who played online casino games was significantly lower after the global pandemic began compared to before it. Not only did players wager less on sports (because there was so little sport to bet on), but they also wagered less on online casino games. Consequently, Auer et al. (2020) argued that there had been no conversion of money spent from sports betting to online casino, at least for the particular online gambling operator that they studied. The same study also found that although online casino gambling did not become more frequent, frequent sports bettors maintained the amount of online casino gambling they engaged in, whereas less frequent sports bettors were more likely to stop gambling altogether. The most intense sports bettors did not appear to play online casino games more or less often when sports betting was not available during the start of the COVID-19 pandemic. Given the lack of studies on the impact of COVID-19 on gambling behavior, the present study investigated the behavior of a sample of online casino gamblers before and after the COVID-19 pandemic was announced in March 2020.

## Method

### Participants

The authors were given access to behavioral tracking data of the entire player base by a large European online gambling operator with several online casino Swedish licenses. The players were therefore exclusively from Sweden. The dataset comprised 133,286 players, and the inclusion criterion was at least one online casino wager from January 1 to May 31 (2020). Online casino games comprised slots games, table games such as roulette and dice, and card games such as blackjack and baccarat. The product portfolio also consisted of sports betting as well as online casino games. The dataset allowed the authors to identify players who had wagered on sports or played online casino games. However, for the underlying analysis, the authors only considered online casino wagers. Online casino gambling activity utilizing daily cross-sectional data was examined over a 5-month period. The number of daily active players and the mean average daily bet across the daily active players were used as measures of gambling intensity. Using the cross-sectional daily data, the authors computed percentile values of the mean amount of money wagered for each day of the 5-month study period across all the active players on each day. Therefore, the study did not track individual online casino gamblers over the 5-month period but tracked the totality of online casino gambling at the site on each day.

### Study Context

Unlike many other countries, Sweden did not introduce a national lockdown to minimize the spread of COVID-19 but resorted to voluntary spatial distancing guidelines (such as working from home where possible, avoiding public transport, and table-only service in restaurants and bars; La Page 2020; Savage 2020). Modeling indicated that Swedes had approximately 30% of the social interactions compared to that prior to the pandemic; that while commercial operations remained open (e.g., shops, gyms, bars, restaurants), footfall was greatly reduced; and that economic spending was reduced as much as in neighboring countries that had a full national lockdown (La Page 2020; Savage 2020).

### Procedure

The study comprised secondary data analysis. The authors computed the number of active online casino players as well as the amount wagered for each day between January 1 and May 31. An active player on a day had to have wagered at least once during that day. The day lasted from midnight to 11:59 pm. In order to identify significant changes in the respective time period, the authors used the Mann-Kendall Test (Yue et al. 2002). The authors also had access to a classification of players into risk categories by the behavioral tracking tool *mentor* (Auer and Griffiths 2015, 2020). Based on behavioral aspects including chasing losses, failed deposits, deposits within sessions, long playing sessions, and other criteria, *mentor* classifies players weekly into low, medium, or high risk. In contrast to risk screening tools such as the Problem Gambling Severity Index (Ferris and Wynne 2001) or the South Oaks Gambling Screen (Lesieur and Blume 1987), *mentor* does not rely on self-reported information about gambling. It solely derives a risk classification from the way the player actually gambles.

## Results

**Fig. 1:**
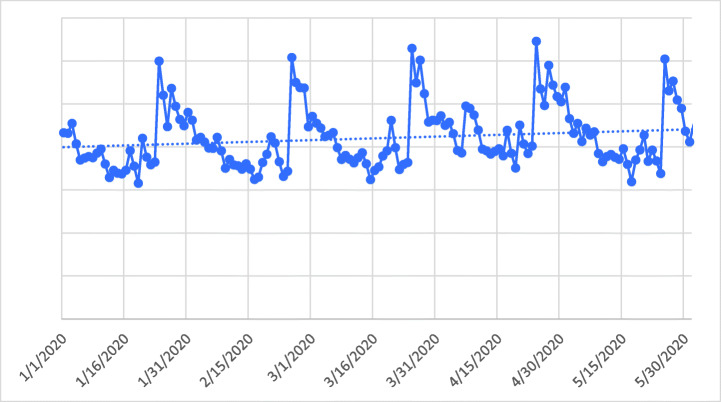
Time series of the number of active online casino players per day (solid line) and the respective linear trend (dashed line). Please note that actual player numbers and gambling spend are not provided for reasons of commercial sensitivity

Figure 1 reports the time series of the number of daily active online casino players as well as the respective linear trend. The average number of active players per day between January 1 and May 31 was 8430. The linear trend was positive and statistically significant according to the Mann-Kendall Test (*Z* = 2.43, *n* = 153 [days], *p* = 0.015), meaning that the number of active online casino players significantly increased over time. There also appeared to be a periodicity which repeated every month each month. The highest number of players can be observed at the end of the months on January 24, February 25, March 25, April 24, and May 25.

**Fig. 2:**
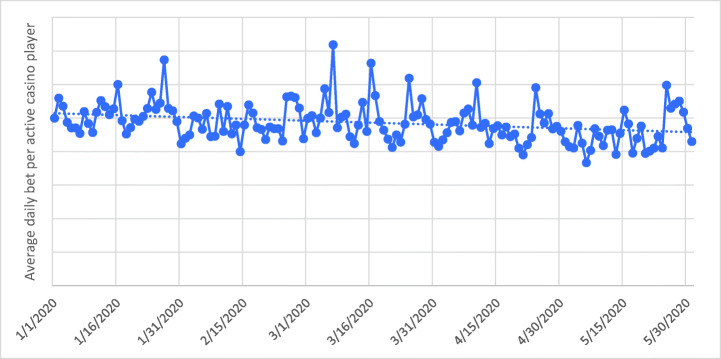
Time series of the mean average daily bet per active online casino player (solid line) and the respective linear trend (dashed line). Please note that actual player numbers and gambling spend are not provided for reasons of commercial sensitivity

Figure 2 reports the time series of the mean average daily bet per active online casino player as well as the respective linear trend. The average daily bet is the ratio between the total amount wagered across all players on that day and the number of players who wagered at least once on that day. The average daily bet per active player between January 1 and May 31 was SEK 9714 (Swedish Krone; approximately €946 and $1132). The linear trend is negative and statistically significant according to the Mann-Kendall Test (*Z* = −4.88, *n* = 153, *p* < 0.001), meaning that the mean average amount of money bet daily significantly decreased over time. There does not appear to be a recurring monthly periodicity. However, there are a few days with larger mean average bet sizes (January 27, March 7, March 16, March 25, April 10, April 24, May 25).

**Fig. 3:**
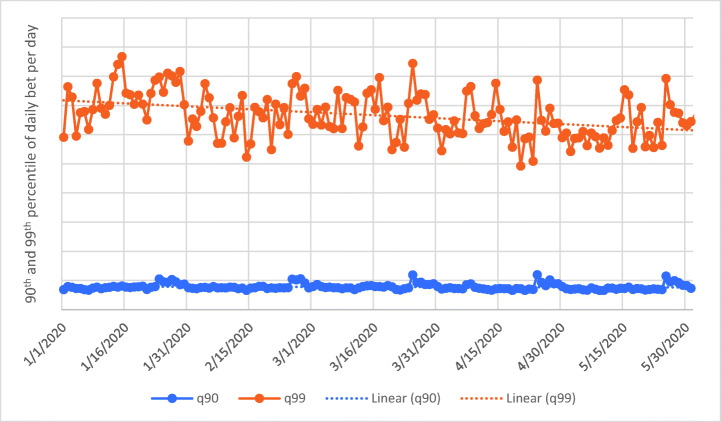
Time series of the 90th and 99th percentiles of daily bet for active online casino players (solid line) and the respective linear trend (dashed line). Please note that actual player numbers and gambling spend are not provided for reasons of commercial sensitivity

**Fig. 4:**
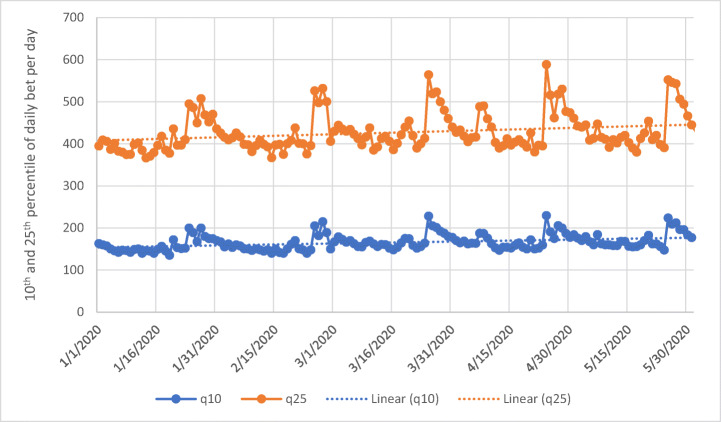
Time series of the 10th and 25th percentiles of daily bet for active online casino players (solid line) and the respective linear trend (dashed line)

In order to investigate whether COVID-19 measures (i.e., measures that promoted spatial distancing and led to more time spent at home) had an effect on subgroups of players, the authors computed the percentiles of the mean average amount of money bet by the online casino players for each day. Figure 3 reports the 90th and the 99th percentiles for each day. These are the values which reflect the online casino players with the highest gambling intensity. The Mann-Kendall Test (*Z* = −5.19, *n* = 153, *p* < 0.001) showed that at the 99th percentile, the mean daily amount of money wagered by online casino gamblers significantly decreased. The mean average daily bet on May 31 was 32% smaller compared to that on January 1. The Mann-Kendall Test (*Z* = −3.50, *n* = 153, *p* < 0.001) showed that at the 90th percentile, the mean average daily amount of money wagered by online casino gamblers also significantly decreased. The authors also computed the 10th and 25th percentiles of the bet across the online casino players for each day. These two values represent the lower spectrum with respect to gambling intensity. Figure 4 reports the two time series which both have a significant positive trend (*Z* = 5.38, *n* = 153, *p* < 0.001; *Z* = 3.70, *n* = 153, *p* < 0.001), meaning that at the 10th and 25th percentile values, low-intensity gambling significantly increased over time. More specifically, the mean average daily amount of money wagered by online casino gamblers on May 31 was 9% larger compared to that on January 1 at the 10th percentile.

The 10th and 25th daily bet quantiles were chosen to be indicative of low-intensity players, and the 90th and 99th daily bet quantiles were chosen to identify high-intensity players. These are to some degree arbitrary given that there is no clear definition of low- or high-intensity players based on specific amounts of money gambled. In order to address this, the Mann-Kendall Test was computed for each daily bet decile as well as the 1st, 5th, 95th, and 99th percentiles. Table 1 reports the Mann-Kendall Test statistic and the resulting *p*-value. A positive test statistic indicates a positive trend and a negative test statistic indicates a negative trend. Up to the 40th percentile, there is a significant increase in daily bet across the observation period. There was no significant change between the 50th and 80th percentiles. From the 90th percentile, there is a significant decrease in the daily bet. This supports the findings based on the 10th, 25th, 90th, and 99th percentiles, respectively. The pattern is consistent with a positive trend in the lower percentiles and a negative trend in the higher percentiles. There did not appear to be a change in daily bet in the midrange.

**Table 1:** Mann-Kendall Test for the time series of daily bet for different percentiles

Daily bet percentile	*Z*	*p*	Significance
1	3.88	<0.001	*
5	6.5452	<0.001	*
10	5.3793	<0.001	*
20	4.2779	<0.001	*
25	3.7015	<0.001	*
30	3.4195	<0.001	*
40	2.9076	0.004	*
50	2.0831	0.037	
60	0.99549	0.32	
75	−0.71921	0.472	
70	−1.3566	0.175	
80	−2.4412	0.014	
90	−3.4952	<0.001	*
95	−4.9628	<0.001	*
99	−5.1879	<0.001	*

*Significant at the *p* < .001 level

**Fig. 5:**
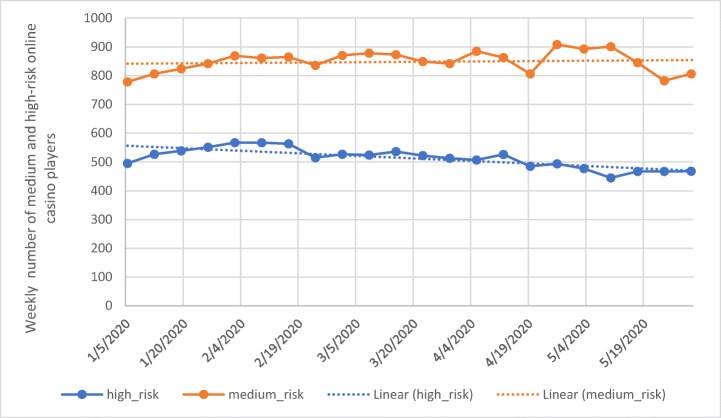
Weekly number of medium- and high-risk online casino players (solid line) and the respective linear trend (dashed line)

The online gambling company which provided the player tracking data also uses the player tracking tool *mentor*, which classifies players weekly according to their gambling-related risk. Figure 5 displays the weekly number of medium- and high-risk online casino players. The number of high-risk players significantly decreased over time (*Z* = −4.2, *n* = 22, *p* < 0.001), while the trend for the medium-risk players was not statistically significant (Z = 0.97, *n* = 22, *p* = 0.33). The authors also computed the average number of playing days across all players active in a specific month. In January, active players in that month (players who placed at least one bet) played on average of 4.4 days. The respective number of active days by online casino players was 4.4 for February, 4.6 for March, 4.5 for April, and 4.5 for May. The Mann-Kendall Test demonstrated that there was no significant trend across time (*Z* = 0.78, *n* = 5, *p* < 0.43) (i.e., the number of days spent gambling on online casino games did not increase over the 5-month period).

## Discussion

The present study investigated online casino gambling behavior of Swedish players from a European online gambling operator before and during the COVID-19 pandemic. The authors specifically focused on online casino players and did not examine sports betting behavior as this was subject to analysis in a previous study (i.e., Auer, Malischnig and Griffiths 2020). Results showed that the online gambling operators’ number of active players increased steadily between January 1 and May 31. The study examined whether active online casino players’ gambling intensity changed over time and computed the mean average daily amount of money bet across all active players for each day between January 1 and May 31 (in 2020). Results showed that the mean average daily bet significantly decreased over time (i.e., online casino players gambled less money as the pandemic continued).

However, the reduced amount of money bet could occur in any player segment. It could be that the high-risk players spent less or that the low-risk players spent less. Theoretically, high-risk players could spend more, and the usually larger segment of low-risk players could spend less. In order to investigate this in more detail, the present study computed the percentiles of the amount bet for each day across all the active players on that day. The 99th percentile reflects the amount bet by the 1% most gambling intense players. The authors posited that this metric would shed light on the behavioral change of the 1% most extreme players each day between January 1 and May 31. The significant negative trend contradicts the assumption that high-intensity online casino players spent more during the start of the COVID-19 pandemic. On the contrary, the mean average amount of money wagered by the 1% most extreme online casino players each day significantly decreased over time. However, at the 10th and 25th percentiles, the mean average daily amount of money wagered by online casino gamblers increased between January 1 and May 31, although it should be noted that the amount of money wagered was relatively small.

Due to confidentiality, the authors cannot report the actual numbers which were bet. However, the magnitude of the change between January 1 and May 31 casts a clearer picture. The average daily bet on May 31 was 9% larger compared to that on January 1 in the 10th percentile. In the 99th percentile, the daily bet on May 31 was 32% lower. This indicates that the drop in amount bet among high-intensity players is much larger than the increase among low-intensity players.

In order to validate the findings regarding the high-intensity online casino players, the authors also computed the number of medium- and high-risk players according to the behavioral tracking tool *mentor*. The analysis indicated that the number of high-risk players significantly decreased between January 1 and May 31. It was also posited that the increased number of active online casino players per day (Fig. 1) and the decreased average daily bet per player (Fig. 1) may have been due to an increased number of newly registered players. This assumes that newly registered players play less intensely than existing players. However, the authors did not have access to players’ registration date, and therefore, it is unclear which players were new and which were existing players. In order to provide some insight on this issue, the average number of playing days for active players in each month was computed. If newly registered players gambled less intensely, they most likely played less frequently. However, the number of playing days did not significantly increase or decrease across the 5-month study period. Therefore, the increased daily bet among low-intensity players is not explained by an increase in newly registered players.

The results of this study are in line with that of Auer, Malischnig, and Griffiths (2020) who (again using tracking data) found that the amount of money bet by online sports bettors significantly decreased during the COVID-19 pandemic (although this was expected given that there was little sport to bet on during the early stages of the pandemic). The study by Auer et al. (2020) also found that online sports bettors did not substitute their sports betting with online casino playing. The present study also found that high-risk gamblers (as identified using the behavioral tracking tool *mentor*) decreased over the 5-month period.

Although tracking data has many benefits, the present study has a number of limitations. First, one of the limitations of account-based data is that the account could be being used by more than one individuals (e.g., a married couple and a parent and one of their adult children living in the same house). However, the total number of shared accounts is likely to be low. Second, all the data were provided by one online gambling operator in Europe with one nationality of gamblers (i.e., Swedish gamblers). Therefore, this is not necessarily representative of online casino gamblers with other operators or of other nationalities. Third, online gamblers are not necessarily loyal to just one online gambling site. Therefore, there may be additional online gambling at other operators’ websites, and the totality of gambling behavior of individuals in the present study (both online and offline) is unknown. Fourth, the dataset comprised cross-sectional daily data of online casino gamblers over a 5-month period rather than individually tracking each online casino gambler. This means that those gambling at specific percentiles in May 2020 were not necessarily the same players gambling at specific percentiles in January 2020.

Findings from the present study suggest there was no increase in the amount of money spent among high-intensity online casino players during the start of the COVID-19 pandemic in Sweden. Overall, the gambling intensity (as measured by the amount of money wagered) of Swedish online casino players significantly decreased during the pandemic. However, low-intensity players’ gambling involvement increased based on the amount of money wagered. This could be due to the fact that more individuals decided to open online casino accounts due to there being more free time available. However, the decrease in overall amount of money spent gambling on online casino games might be because during the pandemic, many individuals suffered financially (i.e., they were unable to earn as much money as they had previously due to the restrictions imposed by the government, although in Sweden, there was no national lockdown). However, many Swedes worked from home during this period, and gamblers may not have wanted to be seen gambling in front of their partner and/or children (as noted by Auer et al. 2020). Additionally, Auer et al. (2020) also speculated that individuals may have spent more “quality time” with their families and/or engaging in home improvement given the increase in hours spent at home (e.g., house and/or garden improvements).

While many different groups (e.g., treatment service providers, policymakers, gambling regulators, and scholars) have claimed that gambling and problem gambling would increase during the pandemic due to more time being spent at home, as well as self-reported studies carried out during the pandemic also suggesting online gambling had increased (Håkansson 2020; Price 2020), evidence from the present study suggests that this is not the case, at least among Swedish online casino gamblers. One of the reasons for this may be because regular gamblers (particularly those that gamble a few times a week or more) tend to have more unreliable self-reports concerning their gambling expenditure based on studies that compared gamblers’ actual gambling behavior using account-based behavioral tracking data versus the same gamblers’ self-reported data utilizing surveys (Auer and Griffiths 2017; Braverman, Tom and Shaffer 2014). In relation to player protection and harm minimization, studies such as the present one suggest that regulators and policymakers should use evidence-based data from all sources rather than rely purely on self-reported data, even when such data are nationally representative. While the use of behavioral tracking data has a number of aforementioned limitations, the main advantage is that the data are objective rather than subjective and are collected from real gamblers, in real time, from real gambling environments.
